# Normal values ​​for ultrasound parameters of the ulnar nerve require homogeneous, healthy cohorts

**DOI:** 10.1186/s42466-023-00254-8

**Published:** 2023-06-29

**Authors:** Josef Finsterer

**Affiliations:** Postfach 20, Vienna, Europe, 1180 Austria

**Keywords:** Ultrasound, Ulnar nerve, Nerve conduction, Normal values

## Letter to the editor

With interest we read the article by Pandal-Fernandez et al. on a study of 76 ulnar nerves from 38 asymptomatic subjects by means of ultrasound [1]. It was found that the diameter of the ulnar nerve as well as the distal and proximal area were larger at the proximal part of the ulnar groove as compared to the distal portion, and even more so in older individuals [1]. In most of the elderly probands, a mild, non-significant reduction of the nerve conduction velocity of < 5 m/s was found at the elbow [1]. The study is excellent, but has limitations that are cause of concerns and should be discussed.

The main limitation of the study is that prior to including subjects in the study, asymptomatic neuropathy was not ruled out. Several neuropathies of the ulnar nerve can be asymptomatic. Subclinical affection of the ulnar nerve can occur with polyradiculitis affecting the 7th and 8th cervical nerve root, plexitis, sulcus ulnaris syndrome, and primary or secondary mono- or polyneuropathy. Therefore, it is crucial that all 38 enrolled subjects had undergone nerve conductions studies prior to enrolment in the study. Probands included in the study should not only have a negative history for neuropathies, but should also have normal proximal and distal nerve conduction studies of the ulnar nerve. The exclusion of subclinical neuropathy is essential before normative data can be collected for ultrasound.

The method section also does not mention what is meant by “asymptomatic” [1]. Do the authors mean no symptoms related to the ulnar nerve or no symptoms related to comorbidities. Were probands with complications in the peripheral nervous system after SARS-CoV-2 infection or vaccination ruled out? No mention is made of current medications, which may also be neurotoxic and therefore could affect the data.

A second limitation of the study is that females predominated with a female-to-male ratio of 2.8:1 [1]. Normative data should be extracted from cohorts with an equal number of females and males. If there are differences between the two groups, different normal values must be generated for both sexes.

Other weaknesses of the study are the small sample size (reference limits are usually derived from a large sample representative of the population of interest), the lack of acceptable sample size estimation considering the variability of the parameters in different populations (age, sex, race, height, body weight, associated health conditions etc.), and lack of evidence that the enrolled subjects were really healthy individuals.

It should be also considered that sleeping habits of the included subjects could affect the evaluation. Those sleeping with a flexed position of the arms may have a propensity to ulnar nerve distension at the ulnar canal as compared to those sleeping in a position with stretched upper limbs.

Additionally, ulnar nerve function and morphology may depend on the vascularisation and perfusion of the ulnar nerve, why it is crucial to know how many of the included patients had classical cardiovascular risk factors, such as arterial hypertension, hyperlipidemia, or smoking, Atherosclerosis of the nerve arterioles may affect function and morphology of a peripheral nerve.

Overall, the interesting study has limitations that call the results and their interpretation into question. Addressing these issues would strengthen the conclusions and could improve the status of the study. Normative data on ultrasound parameters of the ulnar nerve should only be obtained from subjects who actually have normal ulnar nerves. Generation of such cohorts requires not only a medical history devoid of comorbidities associated with neuropathy, but also the exclusion of neuropathy by nerve conduction studies and the exclusion of risk factors associated with neuropathy and atherosclerosis.

## Data Availability

data that support the findings of the study are available from the corresponding author.
